# Transient deformation and swelling of paper by aqueous co-solvent solutions†

**DOI:** 10.1039/d2sm01388f

**Published:** 2023-01-16

**Authors:** C.-L. Wong, S. Wang, S. Karimnejad, M. G. Wijburg, H. Mansouri, A. A. Darhuber

**Affiliations:** a Fluids & Flows Group, Department of Applied Physics, Eindhoven University of Technology The Netherlands a.a.darhuber@tue.nl; b Canon Production Printing Venlo The Netherlands

## Abstract

Inkjet printing inks frequently contain polar liquids of low volatility such as glycerol or poly(ethylene glycols) in addition to the main solvent water. The deposition of these liquids on paper sheets induces swelling of the cellulose fibers, which leads to an overall, anisotropic deformation of the sheet. We characterized the corresponding strain components by means of a grid projection method and white light interferometry. For pure water, most of the hydroexpansion strain vanishes again after drying is complete. However, for aqueous solutions of non-volatile co-solvents, a large fraction of the deformation persists after the water has evaporated. Because swelling occurs only after liquid enters the cellulose fibers, monitoring the dynamics of expansion provides insight into the pore-fiber distribution of co-solvents. The corresponding timescales of pore-fiber transport strongly depend on the co-solvent concentration, as a sufficient quantity of water is needed to plasticize the fiber walls.

## Abstract 2

Inkjet printing inks contain polar liquids of low volatility such as glycerol, besides the main solvent water. Monitoring the expansion of paper substrates after ink deposition provides insight into the pore-fiber distribution of such co-solvents.

## Graphical abstract

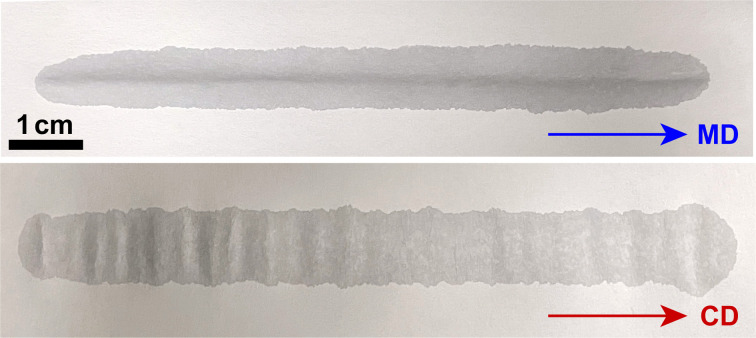

## I. Introduction

The interaction of liquids and specifically water with paper is relevant to *e.g.* paper-making,^1^ food packaging^2,3^ and printing technology.^4^ In the field of inkjet printing, inks are often based on water as an environmentally friendly main solvent.^5,6^ Besides colorants and surfactants,^7,8^ they commonly contain so-called co-solvents – polar liquids such as glycerol or glycols - with a concentration up to 50 wt%.^5^ Their primary purpose is to serve as humectants for preventing inkjet nozzle clogging, but also as anti-cockle and anti-curl agents.^5,9–12^

Generally, polar liquids cause cellulose fibers to swell, leading to an overall deformation of the paper sheet. If gradients of liquid content in the thickness direction of a paper sheet are the dominant driving force, the deformation is called curl.^13–17^ Douezan *et al.*^18^ and Reyssat *et al.*^19^ presented experiments of the curling dynamics of tracing paper deposited on a water surface. Moreover they formulated theoretical models for the bending induced by the differential expansion of paper fibers at different positions depending on the imbibition dynamics of the water in the thickness direction. Leppänen *et al.*^14^ accounted for the anisotropy of the elastic properties of paper as well as its heterogeneity. Lipponen *et al.*^15^ and Erkkilä *et al.*^20^ extended this model by accounting for the elasto-plastic material response of paper.

If the presence of in-plane gradients in liquid content is the dominant mechanism, the paper sheet can undergo a buckling instability.^20,21^ De Böck *et al.*^22^ presented a theoretical analysis of the buckling of paper sheets due to an inhomogeneous moisture distribution based on solutions of the Von Kármán equations. However, they disregarded the sensitive dependence of the elastic properties of paper on the moisture content, which implies that their results are restricted to the case of small excursions in moisture content. Lee *et al.* studied the bending and buckling of paper strips that were supported at their ends and moistened in the center by means of a capillary tube.^23^ Their numerical analysis accounted for the moisture-induced change in elastic properties.

In this manuscript, we studied the deformation of paper sheets after deposition of long lines of aqueous co-solvent solutions, after spray deposition or complete immersion and subsequent drying. We characterized the corresponding strain components in the cross machine direction (CD) and the thickness direction (TD) by means of a grid projection method,^24^ sheet length monitoring and white light interferometry (WLI). For pure water, most of the hydroexpansion strain vanishes again after drying is complete. However, for aqueous solutions of non-volatile co-solvents, a large fraction of the deformation persists after the water has evaporated. Interestingly, this persistent post-drying deformation amplitude first increases with co-solvent concentration up to about 50 wt%, beyond which it decreases. This non-monotonic behavior is attributed to a partial retardation of pore-fiber transport for low water concentrations, as water plays the role of a plasticizer of the cellulose fiber walls.^6^

## II. Materials and methods

### A. Materials and material properties

The co-solvents glycerol, ethylene glycol (EG), tri- and tetra ethylene glycol (TrEG and TEG) were purchased from Sigma-Aldrich and used as received. The molecular weight (*M*_W_), viscosity *μ*_cs_, surface tension *γ*_cs_ and mass density *ρ*_cs_ of the pure co-solvents are listed in Table 1. Aqueous co-solvent solutions were prepared by mixing deionized water (Millipore Direct-Q3 R) and a pure co-solvent at a certain mass ratio in a glass bottle. Masses were measured with a Kern ALT 220-5DAM scale.

**Table 1:** Material properties of pure co-solvents. The values of viscosity *μ*_cs_ and surface tension *γ*_cs_ are given for temperatures of 20 °C and 25 °C, respectively

Co-solvent	Product#	*M*_W_	*μ*_cs_ (mPa s)	*γ*_cs_ (mN m^−1^)	*ρ*_cs_ (kg m^−3^)
Glycerol	449770	92.1	1206	63.5	1258
Ethylene glycol (EG)	324558	62.1	19.8	48.0	1110
Triethylene glycol (TrEG)	90390	150.2	49.0	45.5	1120
Tetra thylene glycol (TEG)	110175	194.2	58.3	44.0	1121

In some experiments we used aqueous solutions of the surfactants sodium dodecyl sulfate (SDS, anionic, *M*_W_ 288, Sigma Aldrich, product number 436143) and Triton X-100 (TX-100, non-ionic, average *M*_W_ 647, Sigma Aldrich, product number T9284). The critical micelle concentration (cmc) of SDS is 0.234 wt%, that of Triton X-100 is 0.019 wt%.

As substrates we used two uncoated paper types from different manufacturers that were both developed for inkjet printing applications.

• paper ‘A’ (DNS High-Speed Inkjet Natural Feel, Mondi, grammage *g* = 80 g m^−2^, thickness *d*_sub_ = 104 μm),

• paper ‘B’ (Z-Plot 650, Ziegler, *g* = 90 g m^−2^, *d*_sub_ = 116 μm, ash content 12%, surface pH 7.3).

The papers are stored in a sealed container until each experiment to reduce the effect of humidity fluctuations.

Nissan developed an empirical model for the dependence of Young's modulus *Y* of paper on its moisture content *θ*_w_ based on the effect of water on the number of inter-fiber hydrogen bonds. This model predicts a drastic reduction of *Y* as *θ*_w_ approaches the maximum holding capacity.^25^ Such a decrease of *Y* with increasing *θ*_w_ has been confirmed by many studies.^23,26–30^ Typically, the value of *Y* for fully wet paper is more than 20 times lower than that of dry paper.^31^

The elastic properties of machine-made paper are anisotropic due to the extensional deformation and tension in the so-called machine direction (MD). Typically the ratio of *Y*_MD_/*Y*_CD_ ranges between 2 and 3, *i.e.* paper is more compliant in the cross-machine direction (CD).^32–39^ This behavior persists upon increasing the moisture content.^26^ Typical values of *Y*_CD_ are in the range of 1.4–4 GPa.^26,35,37,40,41^ The hygroexpansion coefficient is also strongly anisotropic. It is commonly larger in the CD than in the MD direction by a factor typically ranging from 2 to 5.^38,39,42,43^ The hygroexpansion coefficient in the thickness direction (TD) is typically a factor of 7 to 30 larger than in the plane of the paper sheet.^44^ The hydroexpansion coefficients for paper in contact with liquid-phase water have been less intensively studied. Larsson *et al.* spray-deposited water to increase the moisture content of magazine paper.^45^ For an increment Δ*θ*_w_ ≈ 0.04 kg kg^−1^, they found a strain amplitude Δ*ε*_CD_ ≈ 0.14%. Figueiredo *et al.* measured the wet expansion of different paper types^46^ and found values in the range of Δ*ε*_CD_ ≈1.5–3%.

### B. Liquid deposition and optical grid projection

**Fig. 1:**
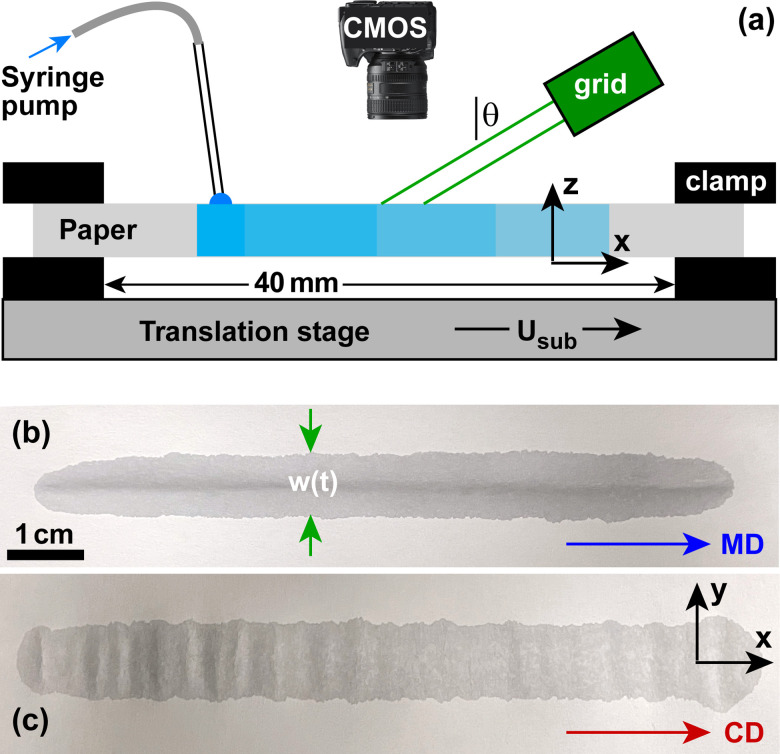
(a) Sketch of the experimental setup. (b and c) Top-view photographs of wet zones deposited *via* substrate motion in the (b) machine direction (MD) and (c) cross direction (CD).

Fig. 1(a) shows a sketch of the setup used for grid projection metrology, a corresponding photograph is available in Fig. 1 of the ESI.† A sheet of paper is suspended about 2 mm above the top surface of a motorized translation stage (Newport, model UTS 100CC). A motorized syringe pump (KD Scientific, model Legato 180) equipped with a gastight syringe (Hamilton, product number 1010, total volume 10 mL) supplies liquid onto the paper through a plastic tube (Hamilton, product number 90618). The tubing orifice was suspended approximately 0.5 mm above the paper surface. The paper is clamped on both sides by two metal plates that have an oblong opening with a width of *w*_clamp_ = 40 mm. A grid projector (Advanced Illumination, model SL191-530IC) is mounted approximately 15 cm above the paper and projects gridlines under an angle of incidence of about *θ* = 40°. A CMOS camera (Thorlabs, model DCC3240M) mounted 35 cm above the paper sheet monitors the displacement and deformation of the projected gridlines. The lateral shift of the projected line corresponding to a certain vertical displacement of the paper sheet was calibrated using a precision labjack (Thorlabs, model number L490) and a digital displacement gauge (Mitutoyo, model number ID-H0530).

Fig. 1(b and c) shows top-view photographs of wet zones deposited *via* substrate motion in the (b) machine direction (MD) and (c) cross direction (CD) of the paper. In both cases the expansion of the paper leads to a buckling instability with bulges oriented along the MD, because both the expansion coefficient and the compliance in the CD is significantly larger than in the MD. In all following experiments, the direction of substrate motion was oriented along the MD. The substrate speed and the volume flow rate of the syringe pump were set to *U*_sub_ = 2 mm s^−1^ and 0.1 mL min^−1^, respectively, unless stated otherwise. The time delay between liquid deposition at the measurement location and the time corresponding to the first frame of the CMOS camera recording (which we denote as *t* = 0) is about Δ*t* ≈ 25 ± 5 s. All experiments were conducted at room temperature and an ambient humidity of (40 ± 10)%.

### C. Sheet length monitoring of square-shaped samples

The combination of line deposition and grid projection allows for convenient monitoring of the time-dependence of the swelling. However, it is restricted to co-solvent contents *θ*_cs_ (kg co-solvent per kg of dry paper) below 0.5 kg kg^−1^ due to rapid lateral imbibition. To reach larger values of *θ*_cs_, we prepared square paper samples of width *w* = 25.4 mm with sides parallel to the MD and CD, respectively, and positioned them on flat polycarbonate plates. A known volume (typically 20–50 μl) of co-solvent solution was deposited homogeneously on the sample by means of a Hamilton digital syringe. In this fashion values of *θ*_cs_ up to about 1.0 kg kg^−1^ could be reached. The swelling causes length expansion, which was measured using an upright microscope (Olympus BX50), a manual translation stage (Newport) and a digital displacement indicator (Mitutoyo, product number ID-H530) with a resolution of 0.5 μm.

### D. Microbead displacement

We positioned a glass microsphere (approximate diameter 200 μm) onto a dry sheet of paper, placed it onto an upright microscope (Olympus, BX50) and focused onto it. Subsequently, EG or glycerol was deposited onto the paper, which caused it to swell in the thickness direction and displace the bead vertically. We measured the change in focus position by means of a z-stage (Kohzu, model number ZM10A-C3C) and a digital displacement indicator (Mitutoyo, product number ID-H530). Conformal contact of the paper strip with the underlying substrate was ensured by slightly bending it into a parabolic shape.

### E. Sample preparation for white-light interferometry

Samples were fabricated by depositing solution droplets on a circular paper sample with a diameter of 12.7 mm. The paper circle was placed on a flat, polished silica or silicon substrate. Samples in which the co-solvent is intended to partially remain within the inter-fiber pores were created with a high concentration of 60 wt%. Samples in which a pore-fiber equilibrium is required were prepared with low concentration solutions of 30 wt% and underwent a post-deposition rehydration treatment with water,^6^ since water accelerates the pore-fiber equilibration. The desired co-solvent content *θ*_cs_ was achieved by deposition of aqueous co-solvent solution droplets of different volumes. Care was taken to prevent excess liquid from spilling over the paper sample. After completion of every deposition and rehydration step, the samples were allowed to dry for at least 45 minutes.

### F. White-light interferometry (WLI)

**Fig. 2:**
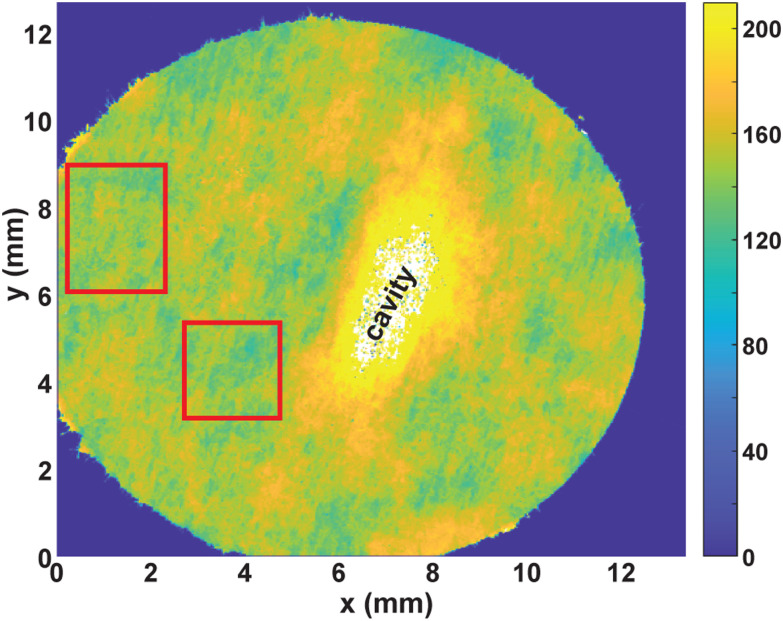
Surface topography of a sample of paper A measured with white-light interferometry. The glycerol content was *θ*_cs_ = 0.9 kg kg^−1^, the substrate a silica wafer. The color bar denotes the surface topography in μm. Red rectangles denote regions for which the average expansion strain is determined.

We used a Bruker NPFLEX white light interferometer with a 5× objective lens and a 0.55× demagnification lens, resulting in a field of view of approximately 2 mm per frame. In order to measure a representative area of the paper samples, we used overlapping regions of interest that the NPFLEX software stitched together, see Fig. 2 for an example. The instrument only records a signal from coordinates where the local slope is smaller than the numerical aperture of the objective lens. For a rough sample such as a paper sheet, this results in a sparse data set. Therefore, we applied a running average over ±5 neighbouring pixels for visualization purposes. Moreover, we fit a plane to the substrate signal (the blue region in Fig. 2) and subtracted it, to eliminate any non-zero tilt of the substrate. In the white region in the center of Fig. 2, the *z*-coordinates of the paper surface were beyond the scan range, due to the presence of a cavity between the paper and the substrate. The height variations outside of the cavity region represented by green and yellow colors amount to approximately ±25 μm. Part of this variation is due to the intrinsic heterogeneity of the paper, another part may be caused by imperfect attachment to the substrate. In our data analysis, we average over 2D regions (indicated by red rectangles in Fig. 2), that are large enough to eliminate the effect of heterogeneity and that do not overlap with the region of non-conformal contact.

## III. Experimental results

We quantify the hydroexpansion in terms of the strain components in the CD1
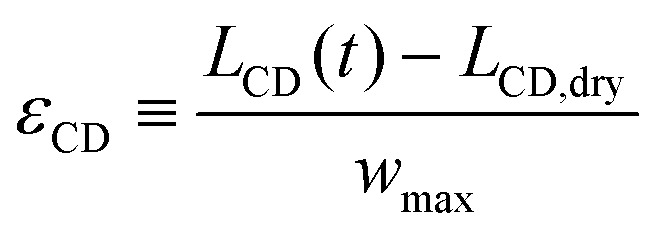
and in the TD2
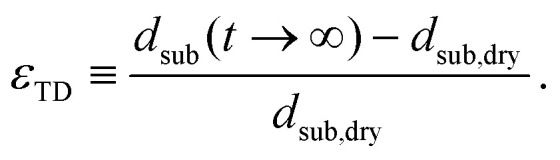
Here, *w*_max_ is the maximum width of the wet zone,3
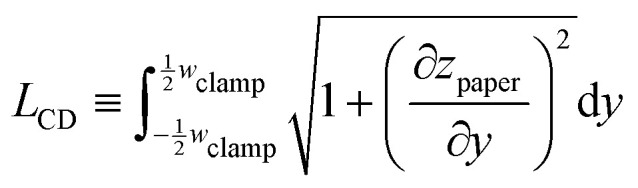
is the length of the paper sample between the clamps and *z*_paper_(*y*,*t*) is the vertical position of the paper surface. We denote the deformation amplitude

4 Δ*z* ≡ *z*_paper_ − *z*_paper,dry_.

The thickness of the paper sheet after co-solvent deposition and after complete water evaporation is denoted *d*_sub_(*t* → ∞), that of the dry paper prior to co-solvent deposition *d*_sub,dry_.

We note that the division by *w*_max_ in eqn (2) corresponds to the notion that primarily the paper in the wet zone expands, whereas the paper that remains dry only deforms to maintain continuity of *z*_paper_(*y*), but without concomitant change in length. Any hygroexpansion due to water evaporating from the wet zone and condensing on adjacent regions of dry paper is disregarded in this fashion. For the practical evaluation of eqn (3), we first smoothed the raw data using a running average, as otherwise the presence of noise will lead to an overestimation of 
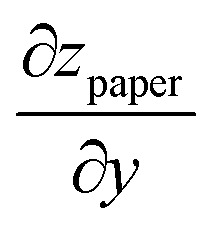
 and thus of *L*_CD_.

### A. Expansion in the cross direction – grid projection

**Fig. 3:**
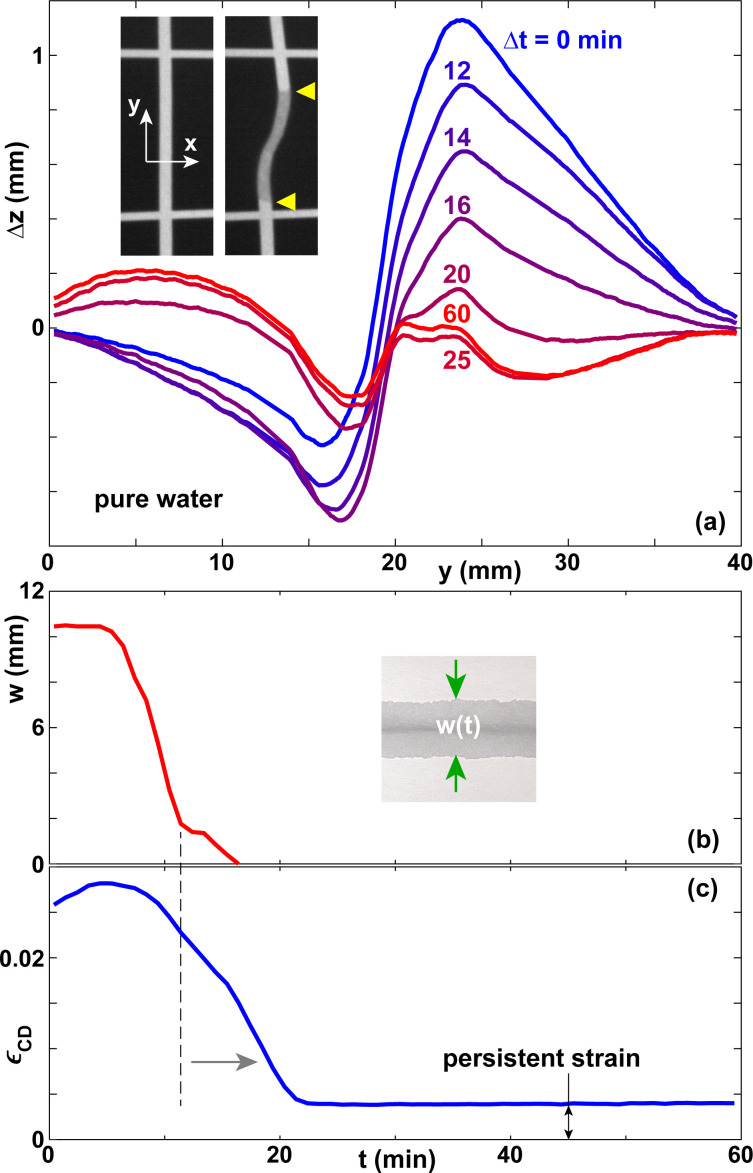
(a) Vertical displacement Δ*z*(*y*) for different times Δ*t* for lines of pure water deposited on paper A. The insets show the projected grid before and about 3 min after deposition. The yellow triangles illustrate the boundaries of the wet zone. The width of the images in the *y*-direction is about 20 mm. (b) Width of the wet zone *w* and (c) lateral expansion strain in the CD direction *ε*_CD_ as a function of time.

Fig. 3 shows the deformation amplitude Δ*z*(*y*), the width of the wet zone *w*, and the expansion strain *ε*_CD_ as functions of time. The curve of *w*(*t*) terminates at *t* ≈ 15 min, due to the evaporation of the water. However, the curve of *ε*_CD_(*t*) reaches a steady state only at *t* ≈ 21 min. This is because upon evaporation the water first depletes the micron-scale inter-fiber pores, which determine the optical appearance of paper.^6^ Most of the water in the fibers is still present and only after it has evaporated as well does *ε*_CD_(*t*) reach a steady state. For pure water, 80–90% of the maximum hydroexpansion amplitude disappears after evaporation is complete. Fig. 3(a and c) shows that the paper sheet does not go back to a flat shape, but a small residual deformation remains, which we denote as persistent strain in Fig. 3(c).

**Fig. 4:**
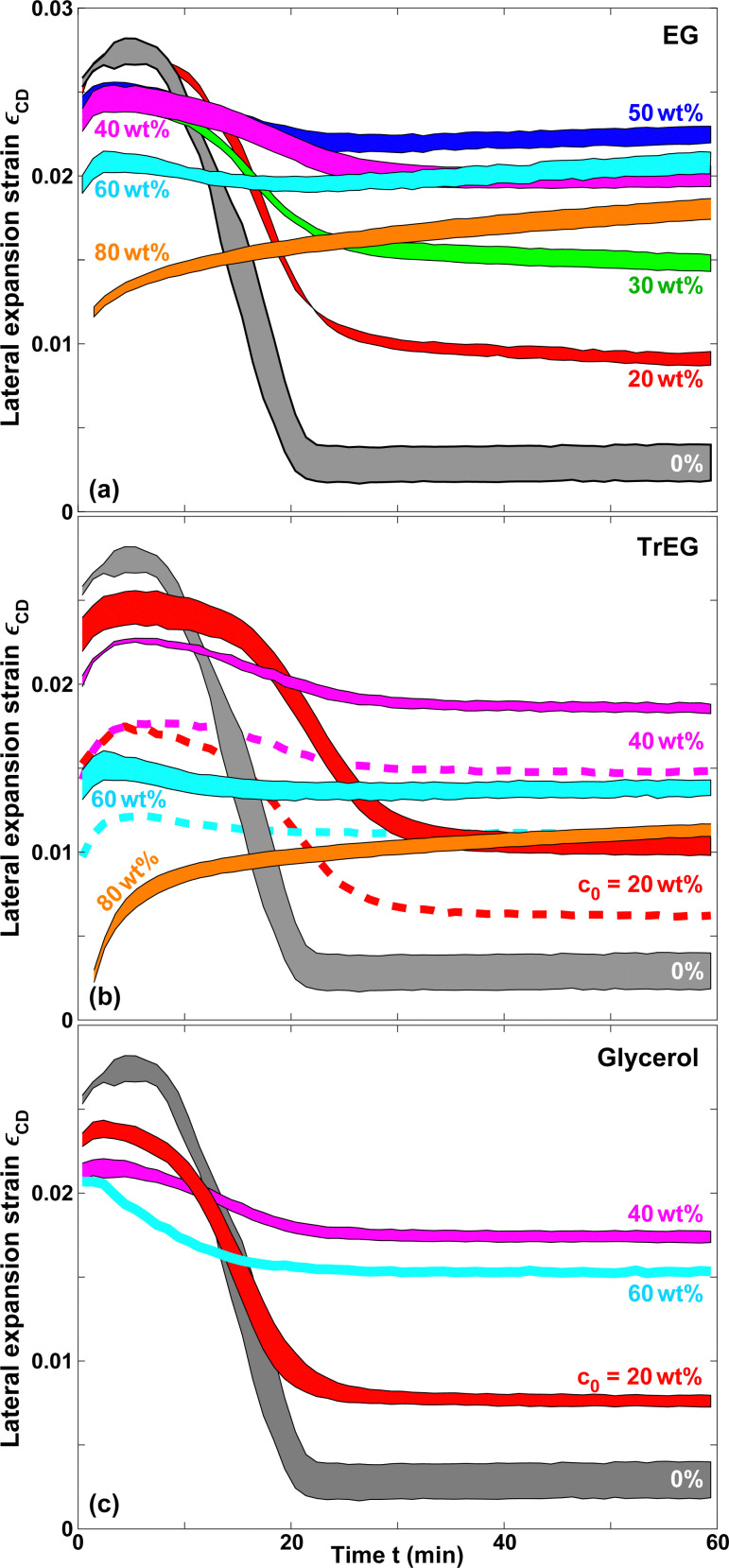
Lateral expansion strain *ε*_CD_ in the cross direction induced by aqueous solutions of (a) EG, (b) TrEG and (c) glycerol as a function of time. Solid lines correspond to paper A, the dashed lines in (b) to paper B.

Fig. 4 presents *ε*_CD_ for EG, TrEG and glycerol solutions of various initial concentrations *c*_0_. In contrast to pure water, the strain does not revert back to almost zero after the water has evaporated, but a large fraction of it persists. This is because the polar co-solvents studied are essentially non-volatile and induce a similar degree of swelling of the cellulose fibers as water, once they have penetrated the fiber walls.^47^

For all co-solvents studied, *ε*_CD_(*t* = 60 min) monotonically increases with increasing initial co-solvent concentration up to a critical value of about 50 wt%. For *c*_0_ exceeding this value, the persistent expansion strain *ε*_CD_(*t* = 60 min) decreases. We attribute this effect to the lack of complete pore-fiber equilibration for high concentration solutions.^6^ EG has the smallest molecular weight of the set and thus suffers less from this effect than TrEG or glycerol. Moreover, the 60 wt% curve for EG has a noticeable upwards slope at *t* = 60 min, which is less pronounced or absent for TrEG and glycerol.

The dashed lines in Fig. 4(b) correspond to experiments with paper B, which generally results in lower values of *ε*_CD_ than paper A (solid lines) by about 15–30%. The bending rigidity^48^ of linear elastic plates scales as *d*_sub_^3^. The thickness of paper B (*d*_sub_ = 116 μm) is approximately 11% larger than that of paper A (*d*_sub_ = 104 μm). The above scaling relation thus would imply a reduction of the deformation amplitude for paper B by approximately 27% compared to paper A. While this is a reasonable agreement, we note that the different expansion amplitudes of the two paper types might also be affected by structural or compositional differences and not just their thicknesses.

#### Effect of surfactants

Fig. 2 of the ESI† shows *ε*_CD_ for aqueous solutions of SDS and TX-100 of different initial concentrations *c*_0_. For both surfactants, the maximum value of *ε*_CD_ as well as the persistent strain *ε*_CD_(*t* ≥ 30 min) are slightly smaller than for pure water. Murali *et al.* found that the maximum holding capacities of papers A and B for surfactant solutions are higher than for pure water by about 12%. Shepherd and Xiao attributed the higher absorption capacity of cellulose-based materials for surfactant solutions to an increased debonding of the fiber network.^49^ We speculate that any debonding might affect primarily *ε*_TD_ rather than *ε*_CD_. It is consistent with the observed slight lowering of the persistent strain.

#### Mode selection

**Fig. 5:**
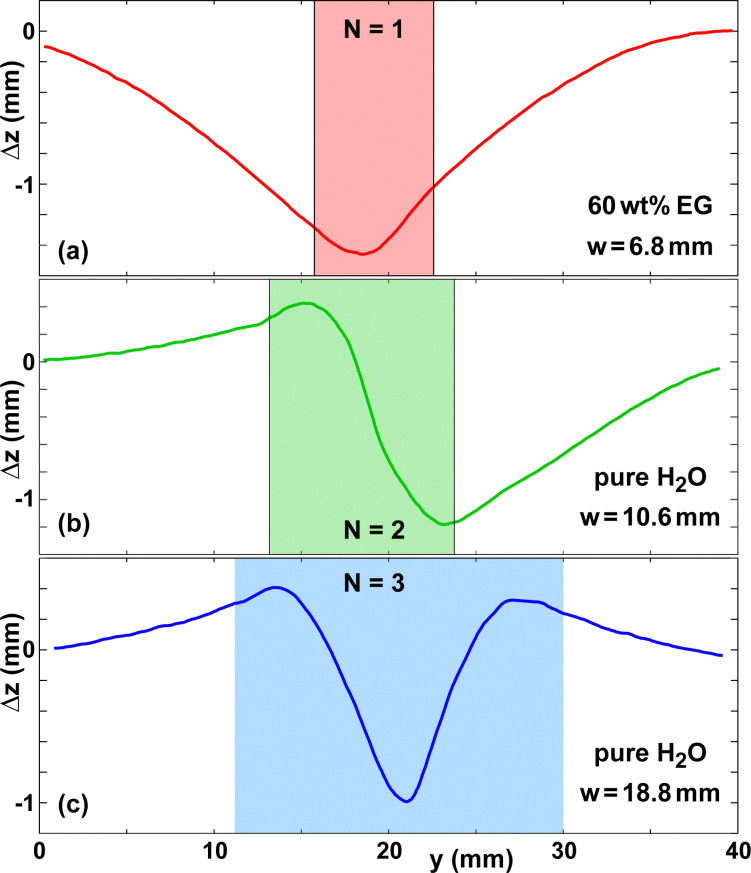
Examples of deformation profiles Δ*z*(*y*) for (a) 60 wt% EG on paper B and (b and c) pure water on paper A. The substrate speed of (c) was *U*_sub_ = 1 mm s^−1^ in contrast to all other experiments.

The shape of the deformed paper sheet can be characterized by the observed number *N* of maxima and minima of *z*_paper_(*y*). We found *N* to range from 1 to 3, with the majority of the experiments resulting in *N* = 2. Fig. 5 shows the deformation amplitude Δ*z* for three illustrative cases. The shaded regions denote the respective wet zones. The most decisive parameter determining *N* is the width of the wet zone. Generally solutions with *c*_0_ = 60 wt% give rise to *N* = 1. Due to the higher viscosity caused by the higher concentration, the width of the wet zone was noticeably smaller. It is clearly visible that the regions of high curvature of Δ*z* are inside the wet zones, which is consistent with Young's modulus of wet paper being much smaller than that of dry paper. The border of the wet zone coincides to good approximation with the location of inflection points in Δ*z*.

#### Co-solvent distributions in pore-fiber equilibrium

**Fig. 6:**
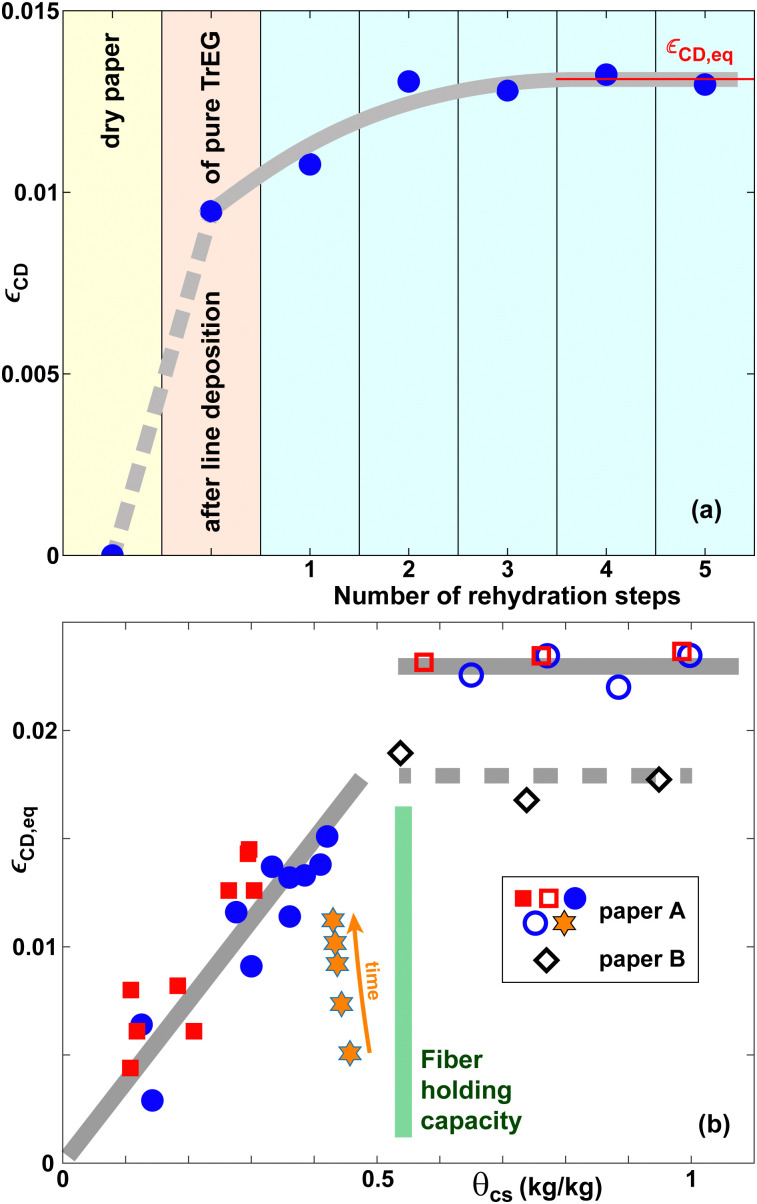
(a) Lateral expansion strain *ε*_CD_ in the CD as a function of the number of rehydration steps for pure TrEG. (b) Equilibrium lateral expansion strain *ε*_CD,eq_ in the cross direction (CD) as a function of *θ*_cs_ for EG (red squares) and TrEG (blue circles, diamonds, hexagrams). The gray lines in (a and b) are guides to the eye. Squares, circles and hexagrams refer to paper A, diamonds to paper B. The orange hexagrams represent selected timesteps of the *c*_0_ = 80 wt% curve in Fig. 4(b).

The pore-fiber transport of high-*M*_W_ co-solvents is greatly retarded in the absence of a sufficient quantity of water, which is needed to plasticize the cellulose fiber walls. For high co-solvent concentrations, the co-solvent therefore primarily resides in the inter-fiber pores after deposition. To facilitate pore-fiber equilibration, we have added pure water after co-solvent deposition by means of an atomizer.^6^ The quantity of water added in each step is approximately equal the quantity of co-solvent solution deposited initially with the syringe pump. Fig. 6(a) shows *ε*_CD_ as a function of the number of water spray deposition steps for pure TrEG. The first data point represents the initial state of dry paper; the second corresponds to *ε*_CD_ 60 min after line deposition, but before any rehydration. The strain amplitude initially increases with increasing number of treatment steps and then saturates at a value *ε*_CD,eq_ for *n* ≥ 2, which we interpret as the co-solvent having reached a state of pore-fiber equilibrium. Fig. 6(b) shows the equilibrium strain values *ε*_CD,eq_ as a function of co-solvent content *θ*_cs_ for EG and TrEG. We have varied *θ*_cs_ by using solutions with different *c*_0_ (line deposition method, solid symbols) or different deposition volumes (sheet length expansion method, open symbols).

The values of *ε*_CD,eq_ increase with *θ*_cs_ for *θ*_cs_ ≲ 0.5 kg kg^−1^ approximately linearly and then saturate. There is essentially no difference in *ε*_CD,eq_ for EG and TrEg to within experimental uncertainty. Our interpretation is that for small quantities of co-solvent, the co-solvent is essentially fully absorbed by the fibers until the maximum holding capacity of the fibers *θ*_HC,fibers_ ≈ 0.5 kg kg^−1^ is reached. For co-solvent quantities beyond this limit, a fraction of co-solvent resides in the inter-fiber pores, where it does not induce any further lateral expansion, however.

The open diamonds in Fig. 6(b) correspond to paper B, all other symbols to paper A. Paper B exhibits expansion strains that are approximately 20% smaller than for paper A, consistent with the behavior found in Fig. 4(b).

The hexagrams in Fig. 6(b) represent selected datapoints from the 80 wt% TrEG curve in Fig. 4(b). The expansion strain increases in time, but after 60 min it is still smaller than the equilibrium value corresponding to the same value of *θ*_cs_. In contrast, the strain value for 50 wt% EG solutions in Fig. 4(a) after 1 hour is essentially equal to the equilibrium value.

### B. Thickness increase – white-light interferometry

**Fig. 7:**
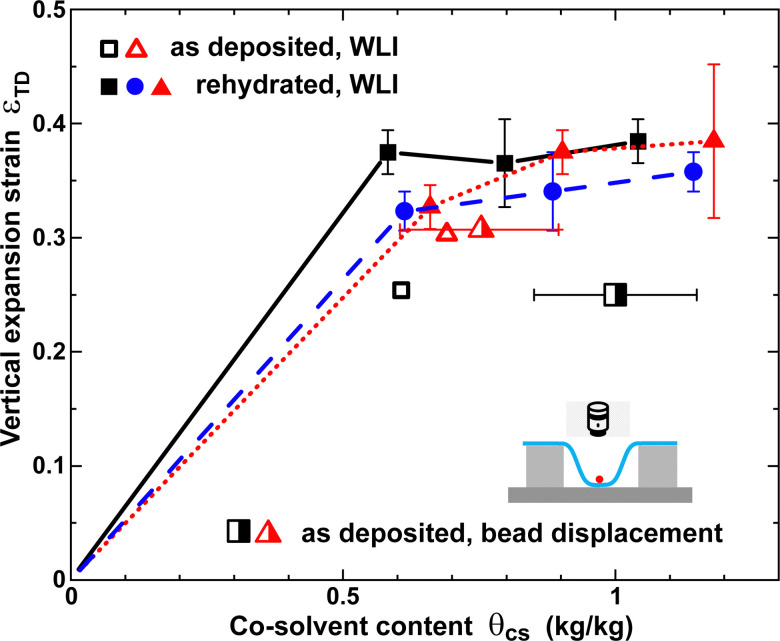
Vertical expansion strain *ε*_TD_ in the TD as a function of co-solvent content *θ*_cs_ for TEG (squares, circles) and glycerol (triangles). Solid squares and triangles refer to paper A and circles to paper B containing equilibrium co-solvent distributions after multiple rehydration treatments. (Half)open symbols refer to paper A and non-equilibrium, as-deposited 60 wt% co-solvent distributions without rehydration steps.

Fig. 7 shows the vertical expansion strain *ε*_TD_ measured as a function of co-solvent content *θ*_cs_. Due to the tendency of paper to wrinkle, which prevents conformal contact with the substrate, we were unable to do reliable measurements for *θ*_cs_ below 0.5 kg kg^−1^. Filled symbols in Fig. 7 correspond to rehydrated samples and thus equilibrium co-solvent distributions. Open symbols in Fig. 7 represent measurements for non-rehydrated, as-deposited, non-equilibrium 60 wt% co-solvent distributions, which result in smaller swelling amplitudes, consistent with the behavior observed in Fig. 4. Semi-filled symbols refer to non-rehydrated, as-deposited, co-solvent distributions of glycerol (triangle) and EG (square) measured using microbead displacement.

Above *θ*_cs_ > 0.5 kg kg^−1^, *ε*_TD_ is only weakly dependent on *θ*_cs_, consistent with the in-plane behavior shown in Fig. 6(b). It is evident that the strain amplitude in the TD is approximately a factor of 20 larger than in the CD. Cellulose fibers can expand easily in the TD, because they have been strongly compressed in this direction during the paper fabrication process. There is little difference between TEG and glycerol concerning the asymptotic value of *ε*_TD_. There is a roughly 10% difference in the swelling amplitudes for TEG in papers A and B.

### C. Expansion in the machine direction

In the MD, we observed an expansion strain of about 0.2% one hour after deposition of pure TrEG onto dry NF paper. After repeated water spray treatments, however, we observed a net shrinkage relative to the initial, dry state of approximately 0.2%. This is consistent with the hysteretic behavior observed for paper subject to cycling of the ambient relative humidity.^39,50,51^

## IV. Discussion

### A. Mode selection

An interesting question pertains to the direction of surface deformation of the *N* = 1 deformation mode. There are three competing mechanisms at play. The first is gravity, which induces a downwards deformation. The second is the elastocapillary deformation of the paper sheet due to the interfacial tension and capillary pressure of the deposited droplet, which both lead to a deformation towards the underside of the paper sheet.^52–55^ This deformation mechanism is facilitated by the drastic decrease of the elastic modulus of paper with increasing moisture content. The gravitational force scales with *ρ*_liq_*V*_drop_*g*, where *g* is the gravitational acceleration. In contrast, the surface tension force is proportional to the droplet footprint radius and scales as *γ*_liq_*V*_drop_^1/3^. Thus, for small droplet volumes, the influence of gravity becomes negligible compared to elastocapillary effects.

The third mechanism is related to the existence of gradients of the moisture content or liquid content in the thickness direction of the paper sheet. Holmes *et al.*,^17^ Samy *et al.*^56^ and Sun *et al.*^57^ reported on experiments regarding the deposition of solvent droplets on polymer sheets. The solvent swells the top part of the polymer which induces a curvature corresponding to an upwards displacement. This is the same mechanism as responsible for the curl of paper sheets observed by Douezan *et al.*^18^ and Reyssat and Mahadevan.^19^

For co-solvent solutions with low concentration *c*_0_, the imbibition time to bridge the thickness of the paper sheet is short for the two paper types studied, such that vertical gradients in liquid content exist only for a brief time span. In contrast, the elastocapillary and gravitational force due to the presence of the droplet may act for a longer time. Consequently, we expect a bias towards a downwards displacement for low *c*_0_. For higher *c*_0_, vertical gradients persist for a longer time due to the higher viscosity, which may favor an upwards deformation for *N* = 1.

We performed a systematic study of the paper deformation after co-solvent deposition. However, we did not observe a clear trend concerning the direction of deformation, which may point at the influence of random heterogeneities in the paper structure.^58–61^

### B. Pore-fiber distribution of co-solvents

Upon imbibition of liquid into a porous medium, the liquid fills first the largest pores and progressively enters the smaller ones owing to their lower permeability. Paper consists of cellulose fibers with typical intra-fiber pore sizes of approximately 1 to 100 nm and typical inter-fiber pore dimensions in the micron range. This implies that for an equilibrium distribution, characterized by a uniform capillary pressure, a liquid preferentially resides in the fibers, until they are saturated, *i.e.* completely filled. Further addition of liquid will also populate the micron-sized pores. The timescales relevant to this pore-fiber equilibration process, however, sensitively depend on the *M*_W_ of the liquid, because the fiber walls act as a barrier. For swellable polymer membranes in contact with aqueous solutions, it is well known that the concentration of water has a large impact on the effective transport rate of large *M*_W_ solutes through the membrane, because it plasticizes the polymer.^62–64^ We hypothesize that the same effect governs the pore-fiber transport dynamics of co-solvent solutions in paper.

Stone and Scallan developed a solute exclusion technique for probing the accessibility of intra-fiber pores to molecules of increasing *M*_W_.^65,66^ Typical cut-off values of the solute *M*_W_ are in the range 250–5000,^67–69^ therefore we conclude that the co-solvents we used can penetrate the fiber walls given enough time. However, without rehydration the co-solvents are unable to enter the fibres to the same extent in the time available between liquid deposition and completion of drying, compared to rehydrated samples. As only the liquid fraction in the fibers contributes to the expansion of the paper, the expansion strain provides information about the current pore-fiber distribution of the co-solvent. In ref. 6 we had studied the light transmittance *I*/*I*_0_ of co-solvent distributions in paper sheets. We found that light scattering is primarily sensitive to the co-solvent content in the inter-fiber pores. Therefore, monitoring *ε*_CD_ is a complementary technique for characterization of co-solvent distributions, as it is sensitive primarily to the co-solvent content in the intra-fiber pores. In the following subsection, the consistency of the results from strain and transmittance measurements is evaluated.

### C. Quantification of pore-fiber distributions

**Fig. 8:**
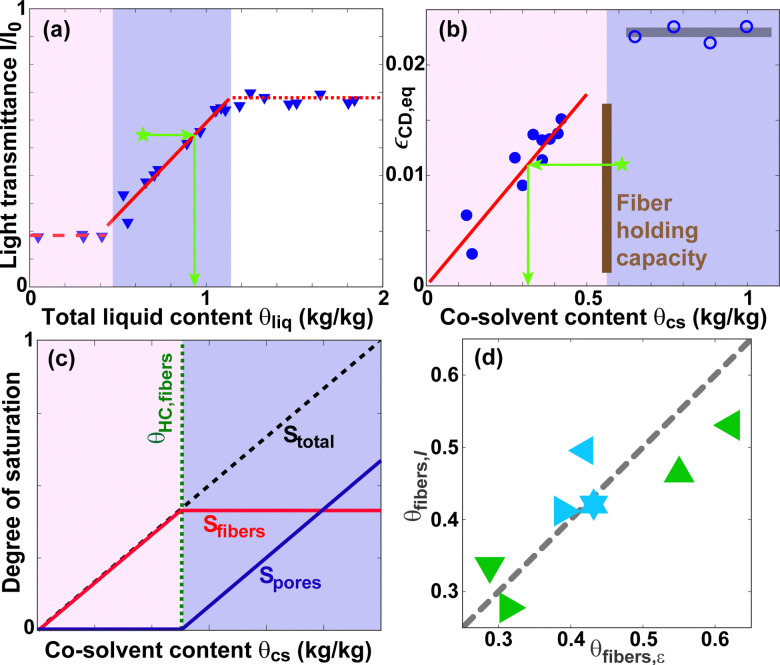
(a) Equilibrium light transmittance *I*/*I*_0_ data for TEG in paper A from ref. 6. The solid red line is a linear fit according to eqn (5). (b) Equilibrium expansion strain *ε*_CD,eq_ for TrEG in paper A. The red solid line is a linear fit according to eqn (6). (c) Simplified model of the equilibrium co-solvent distributions in the intra- and inter-fiber pores of paper. (d) The intra-fiber co-solvent content *θ*_fibers,*I*_ obtained from light transmittance data as a function of the intra-fiber content *θ*_fibers,*ε*_ determined from expansion strain data for the samples listed in Section IV of the ESI.† The dashed line corresponds to *θ*_fibers,*I*_ = *θ*_fibers,*ε*_.

Fig. 8(a) depicts the equilibrium transmittance *I*/*I*_0_ as a function of TEG content of paper A.^6^*I*/*I*_0_ is constant at low *θ*_cs_ ≲ 0.5, followed by an approximately linear increase in the range 0.5 ≲ *θ*_cs_ ≲ 1.1. In contrast, the equilibrium expansion strain *ε*_CD,eq_ is approximately a linear function of *θ*_cs_ for *θ*_cs_ ≲ 0.5, after which is reaches a plateau value [see Fig. 8(b)]. Thus, the equilibrium co-solvent distribution may be approximated as though the co-solvent resides exclusively in the intra-fiber pores at low *θ*_cs_. When *θ*_HC,fibers_ is exceeded, the inter-fiber pores start to fill. This simplification is visualized in Fig. 8(c) in terms of the degrees of saturation *S* ≡ *θ*_cs_/*θ*_cs,max_ of the intra- and inter-fiber pores. Here, *θ*_cs,max_ is the total holding capacity of the paper substrate.

The solid red line in Fig. 8(a) corresponds to the following linear fit of the relation between (*I*/*I*_0_)_eq_ and *θ*_cs_

5 (*I*/*I*_0_)_eq_ = 0.66*θ*_liq_ − 0.07 = 0.66*θ*_cs_/0.95 − 0.07.

Co-solvents are typically hygroscopic liquids, which contain a certain equilibrium quantity of water depending on the ambient humidity. Therefore, the total liquid content is given by *θ*_liq_ = *θ*_cs_ + *θ*_w_. For TEG and RH = 30%, to good approximation *θ*_liq_ = *θ*_cs_/0.95.^70^ The red line in Fig. 8(b) corresponds to the following linear fit of the relation between *ε*_CD,eq_ and *θ*_cs_

6 *ε*_CD,eq_ = 0.0347*θ*_cs_.

For a given non-equilibrium data set *θ*_cs,neq_, (*I*/*I*_0_)_neq_, and *ε*_neq_, a fraction of the co-solvent is in the intra-fiber pores while another fraction is in the inter-fiber pores. Therefore, *θ*_cs,fibers_ can be obtained by inverting eqn (6), which describes the strain corresponding to a certain intra-fiber content,

7 *θ*_cs,fibers_ = *ε*_neq_/0.0347.

The corresponding co-solvent content in the inter-fiber pores *θ*_cs,pores_ is then given by

8 *θ*_cs,pores_ = *θ*_cs,neq_ − *θ*_cs,fibers_.

Similarly, the inter-fiber co-solvent content *θ*_cs,pores_ can be obtained by inverting eqn (5), which describes the transmittance corresponding to a certain inter-fiber pore content,

9 *θ*_cs,pores_ = 0.95[0.07 + (*I*/*I*_0_)_neq_]/0.66 − *θ*_HC,fibers_

and correspondingly

10 *θ*_cs,fibers_ = *θ*_cs,neq_ − *θ*_cs,pores_.

To determine *θ*_HC,fibers_, we define Δ*θ* as the difference in the intra-fiber content calculated from the transmittance and strain data, respectively

11 Δ*θ* = |*θ*_cs,fibers,*ε*_ − *θ*_cs,ibers,*I*_| = |*θ*_cs,pores,*I*_ − *θ*_cs,pores,*ε*_|.

After conducting expansion and transmittance measurements for eight samples (see Fig. 3 in the ESI†), the acquired *θ*_cs,neq_, *ε*_neq_ and (*I*/*I*_0_)_neq_ for each sample are inserted into eqn (7)–(11). The resulting curves of Δ*θ* as a function of *θ*_HC,fibers_ are provided in Fig. 4 of the ESI.† A minimum in Δ*θ* is found at *θ*_HC,fibers_ = 0.56. Inserting this value into eqn (9) and (10) allows the calculation of *θ*_cs,fibers_ and *θ*_cs,pores_ for each sample. The results are visualized in Fig. 8(d) and can also be found in Table 1 in the ESI.† The uncertainty in the quantification is related to their distance from the diagonal line representing *θ*_fibers,*I*_ = *θ*_fibers,*ε*_. The uncertainty amplitude of Δ*θ* at *θ*_HC,fibers_ = 0.56 in Fig. 4 of the ESI† is 0.09, which corresponds to a quantification uncertainty of ±0.05, which is small compared to *θ*_HC,fibers_.

Consequently, the intra- and inter-fiber co-solvent content can be quantified consistently using either mechanical or optical data. Since the transmittance can be measured locally (*i.e.* with high position resolution), whereas the strain is an integral property of an entire sample, *I*/*I*_0_ provides more information.

### D. Hysteresis and history dependence of paper properties

In this subsection, we would like to comment on the validity and consistency of our results, which is intrinsically limited by paper being a history-dependent material. The processes of coming into contact with liquid-phase water and subsequent drying induce irreversible changes not only in the fibrous network structure of the paper sheet but also in the internal conformation of the fibers.^71^ One related aspect is the breakage of inter-fiber hydrogen bonds upon water imbibition and the formation of new hydrogen bonds upon water evaporation. One manifestation thereof is the relaxation of the stress built into paper by its fabrication process upon water immersion, which accounts for the net shrinkage in the MD discussed in Section III.C. Changes of the internal fiber structure are related to *e.g.* hornification^72–75^ and microfibril coalescence.^76^

The comparison of as-deposited samples with samples that have undergone multiple rehydration steps in Fig. 6 and 7 needs to be seen in the perspective of these phenomena. However, we believe that our method and our results are nevertheless valid and useful, because the irreversible and hysteretic strain amplitudes are typically much smaller than the expansion strain amplitudes we observe.^77^ An example is the persistent strain indicated in Fig. 3(c), which is approximately a factor of 10 smaller than the typical expansion strain amplitudes visible in Fig. 4 and 6.

## V. Summary and conclusions

We have studied the anisotropic swelling of paper sheets after deposition of aqueous co-solvent solutions using four different experimental methods. Given the high elastic compliance of wet paper, optical grid projection has the advantage of being contactless. In contrast, in white-light interferometry, microbead displacement and sheet length monitoring, the paper is in contact with an underlying solid substrate.

While the expansion in the cross machine direction (CD) was found to be on order of 2.5%, it amounts to approximately 40% in the thickness direction. Pure water gives rise to a persistent CD expansion strain of about 0.3% after drying, which rises up to 2.4% for solutions of non-volatile co-solvents that remain in the paper after the water has evaporated.

We found that the expansion strain not only depends on the quantity of co-solvent deposited, but also on the history of the sample, *e.g.* the initial co-solvent concentration in the solution. We ascribe this history dependence to the sensitivity of the co-solvent transport through the fiber walls to the concentration of water, which is needed to plasticize the fiber walls before molecules of larger molecular weight can pass through swiftly.

Co-solvents induce swelling primarily when they enter the intra-fiber pores, whereas they affect the optical light transmission primarily when they reside in the inter-fiber pores.^6^ Therefore, monitoring mechanical expansion and light transmission are two complementary methods for characterization of pore-fiber distributions of a co-solvent.

## Conflicts of interest

There are no conflicts of interest to declare.

## Supplementary Material

SM-019-D2SM01388F-s001
