# Supplementary material for: Transient deformation and swelling of paper by aqueous co-solvent solutions
Source: Soft Matter. 2023 Jan 16;19(6):1202–11. doi: 10.1039/d2sm01388f (PMC9906801; doi:10.1039/d2sm01388f)
Supplement: SM-019-D2SM01388F-s001 [file SM-019-D2SM01388F-s001.pdf]

Electronic Supplementary Material (ESI) for Soft Matter.
This journal is © The Royal Society of Chemistry 2023

Supplementary Information for manuscript: Transient deformation and swelling of paper by aqueous co-solvent solutions

C.-L. Wong,[1] S. Wang,[1] S. Karimnejad,[1] M. G. Wijburg,[1] H. Mansouri,[2] and A. A. Darhuber[1]
[1] *Fluids & Flows Group, Department of Applied Physics, Eindhoven University of Technology*
[2] *Canon Production Printing, Venlo, The Netherlands*

(Dated: 9 January 2023)

I. EXPERIMENTAL SETUP

Figure 1 presents a photograph of the optical grid projection setup. A sheet of paper is suspended about

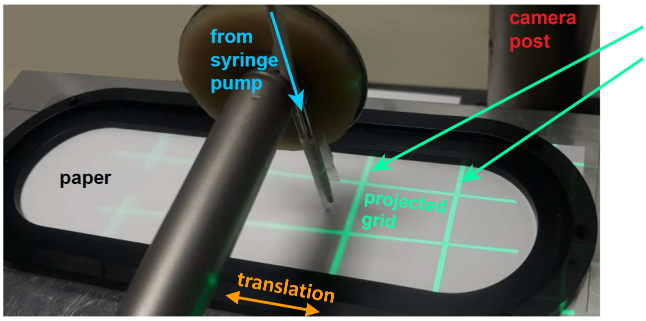

FIG. 1. Photograph of the optical grid projection setup.

2 mm above the top surface of a motorized translation stage (Newport, model UTS 100CC). A motorized syringe pump (KD Scientific, model Legato 180) equipped with a gastight syringe (Hamilton, product number 1010, total volume 10 ml) supplies liquid onto the paper through a plastic tube (Hamilton, product number 90618). The tubing orifice was suspended approximately 0.5 mm above the paper surface. The paper is clamped on both sides by two metal plates (black in Fig. 1) that have an oblong opening with a width of $w_{\text{clamp}} = 40$ mm. A grid projector (Advanced Illumination, model SL191-530IC) is mounted approximately 15 cm above the paper and projects gridlines under an angle of incidence of about $\theta = 40^o$. A CMOS camera (Thorlabs, model DCC3240M) mounted 35 cm above the paper sheet monitors the displacement and deformation of the projected gridlines.

II. EFFECT OF SURFACTANTS

Figure 2 shows ϵ_{CD} for aqueous solutions of SDS and TX-100 of different initial concentrations c_0. For both surfactants, the maximum value of ϵ_{CD} as well as the persistent strain $\epsilon_{\text{CD}}(t \geq 30\,\text{min})$ are slightly smaller than for pure water.

III. INFLUENCE OF AMBIENT HUMIDITY FLUCTUATIONS

The relative humidity affects our experiments in two ways: 1) it determines the evaporation time-scale, i.e. the drying time; 2) it affects what we consider the 'dry' dimensions of the paper sheet, i.e. $L_{\text{sub,dry}}$ and $d_{\text{sub,dry}}$ in Eqs. (1,2), and thus the expansion strain amplitudes. However, considering typical moisture sorption isotherms of paper, for a 10% change in relative humidity (e.g. from 40% to 50%), the equilibrium moisture content changes by 1 − 2 wt%, i.e. 0.01 − 0.02 kg/kg, whereas the liquid content we add is on order of 100 wt% or 1 kg/kg. Also the fiber holding capacity $\theta_{\text{HC,fibers}} \approx 0.56$ kg/kg is much higher. Therefore, we neglect the effect of the ambient

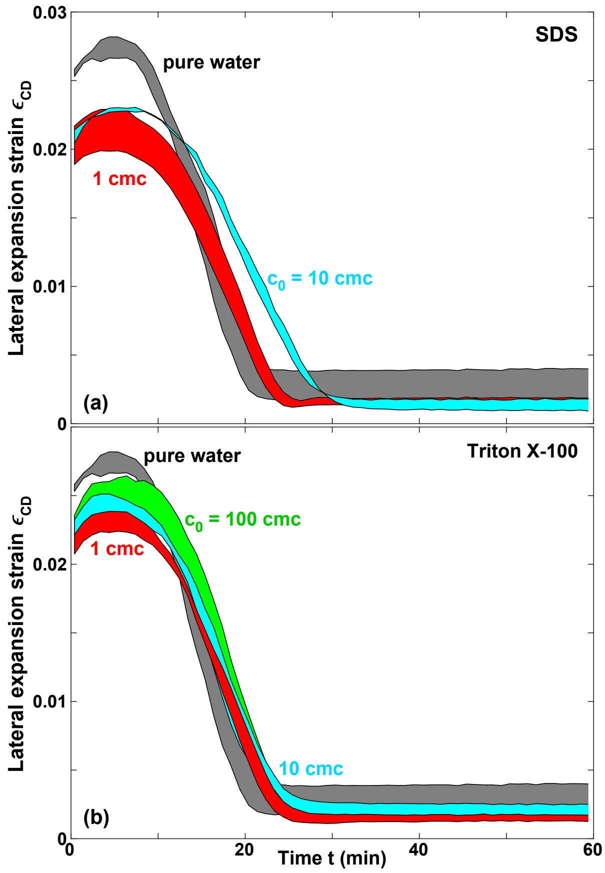

FIG. 2. Lateral expansion strain ϵ_{CD} in the cross direction induced by aqueous solutions of (a) SDS and (b) Triton X-100 as a function of time.

humidity fluctuations.

IV. QUANTIFICATION OF PORE-FIBER CO-SOLVENT DISTRIBUTIONS

We have prepared 8 non-equilibrium samples by depositing TrEG solutions on sheets of paper A. The first column of Tab. I gives the initial concentration c_0 and the overall co-solvent content $\theta_{\rm cs}$. For these samples we have measured both the CD expansion strain and the light transmittance. The light-green and light-blue triangles in Fig. 3 denote these non-equilibrium data plotted together with the corresponding equilibrium curves.

Figure 4 shows $\Delta\theta$ of the 8 samples, from which the best-fit value of $\theta_{\rm HC,fibers} = 0.56$ follows. Using this value, the inter- and intra-fiber co-solvent contents of the 8 samples are presented in Tab. I.

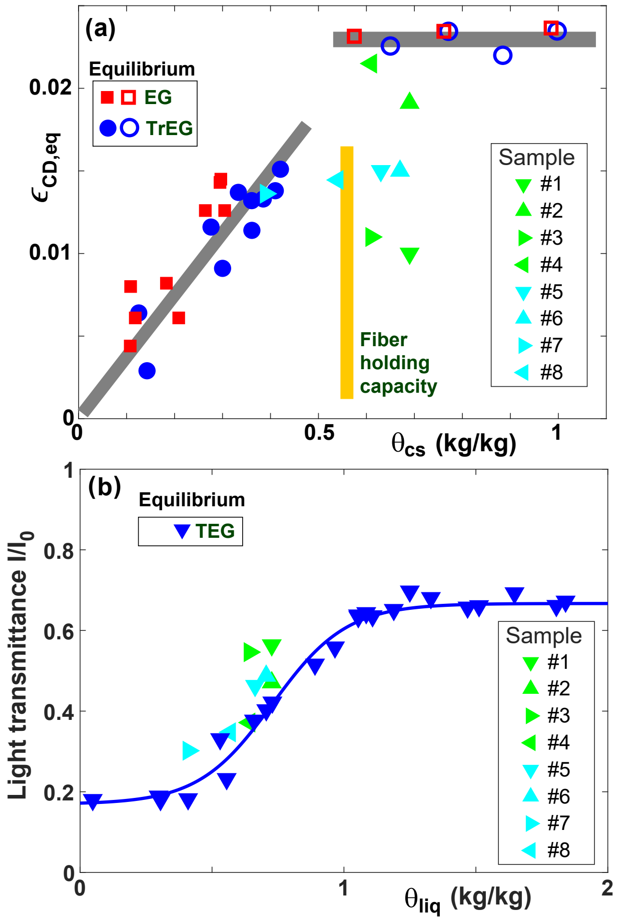

FIG. 3. (a,b) CD expansion strain and light transmittance of 8 non-equilibrium samples (light-green and light-blue triangles) plotted plotted together with the corresponding equilibrium data.

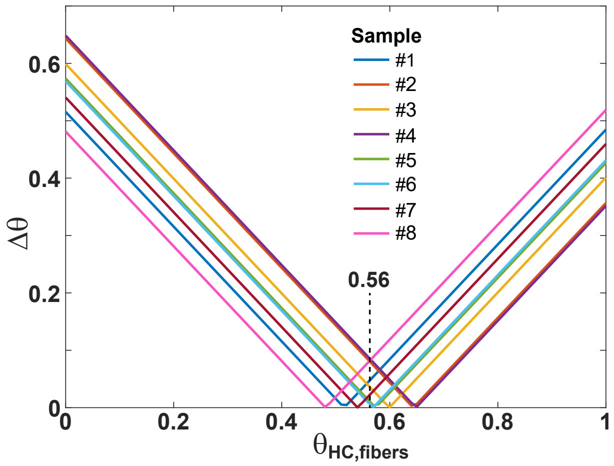

FIG. 4. $\Delta\theta$ as a function of the fiber holding capacity for samples #1 to #8. An overall minimum is found at $\theta_{\mathrm{HC,fibers}} = 0.56$.

Sample	c_0 [wt%]	θ_{cs}	$\theta_{\mathrm{fibers},\epsilon}$	$\theta_{\mathrm{fibers},I}$	$\theta_{\mathrm{pores},\epsilon}$	$\theta_{\mathrm{pores},I}$
#1	100	0.69	0.29	0.33	0.40	0.36
#2	100	0.69	0.55	0.47	0.14	0.22
#3	100	0.61	0.32	0.28	0.29	0.33
#4	100	0.61	0.62	0.53	-0.01	0.08
#5	60	0.63	0.43	0.42	0.20	0.21
#6	60	0.67	0.43	0.42	0.24	0.25
#7	60	0.39	0.39	0.41	0.00	-0.02
#8	60	0.54	0.42	0.50	0.12	0.04

TABLE I. Calculated pore and fiber liquid contents (units kg/kg) of the 8 non-equilibrium samples (TrEG in paper A).
